# Editorial: Therapeutic potential of mesenchymal stem cells in organ and tissue regeneration

**DOI:** 10.3389/fbioe.2023.1333281

**Published:** 2023-11-30

**Authors:** Silvia Barbon, Antara Banerjee, Laura Perin, Raffaele De Caro, Pier Paolo Parnigotto, Andrea Porzionato

**Affiliations:** ^1^ Department of Neuroscience, Section of Human Anatomy, University of Padua, Padua, Italy; ^2^ Foundation for Biology and Regenerative Medicine, Tissue Engineering and Signaling—TES Onlus, Padova, Italy; ^3^ Faculty of Allied Health Sciences, Chettinad Academy of Research and Education (CARE), Chettinad Hospital and Research Institute (CHRI), Chennai, India; ^4^ GOFARR Laboratory, Children’s Hospital Los Angeles, Division of Urology, Saban Research Institute, Keck School of Medicine, University of Southern California, Los Angeles, CA, United States

**Keywords:** mesenchymal stem cells, preclinical studies, clinical trials, tissue regeneration, organ regeneration, cell therapy

In recent decades, the biomedical applications of adult mesenchymal stem cells (MSCs) have increasingly intrigued researchers and clinicians. These cells can be easily isolated from a variety of tissue sources, such as bone marrow (Di Liddo et al., 2014; Dzobo, 2021), adipose tissue (Kuhbier et al., 2010; Al-Ghadban et al., 2022), umbilical cord (Jothimani et al., 2020), synovium (Li et al., 2020), and peripheral blood (Di Liddo et al., 2016; Barbon et al., 2018; Barbon et al., 2021), as well as perinatal tissues (Kulus et al., 2021). These cells exhibit multi-differentiative and trans-differentiative potential (Banerjee A et al., 2022) in accordance with the specific requirements of functional organ or tissue regeneration. Additionally, MSCs lack the expression of histocompatibility complexes and immune-stimulating molecules, and as they are not detected by host immune surveillance, their use reduces graft rejection after transplantation. For these reasons, MSCs have turned out to be competent candidates in tissue engineering (Han et al., 2019; Barbon et al., 2022).

Several studies have explored the potential of MSC-based therapies for tissue regeneration, with encouraging outcomes at both the preclinical and the clinical level. Despite rapid advancements in the field, deeper knowledge regarding the mechanism of action of MSCs in tissue repair and regeneration is required. Thus, this Research Topic collects and promotes papers focusing on the preclinical and translational investigation of MSCs in relation to various therapeutic aspects of organ or tissue regeneration. A total of six papers have been published, which are highlighted here.

The use of perinatal tissues for isolation of MSCs enables the use of less invasive extraction procedures and ensures greater availability of the cell source. Using GMP practices and strict criteria for compatible donors, placental tissue can be safely cryopreserved for long-term storage as an off-the-shelf source for production of therapeutic stem cells (Antoniadou and David, 2016). Navakauskienė et al. investigated the effects of cryopreservation of human placental tissue on the properties of placental MSCs, comparing cells extracted from fresh (native) and cryopreserved (cryo) placenta samples. The study demonstrated that cryopreservation did not affect the proliferation, energy profile, surface marker expression, or multipotent differentiation capacity of MSCs. Moreover, native and cryo placental MSCs expressed only slight variations in epigenetic profile. According to transcriptome analysis, the upregulation of early-senescence state-associated genes in placental MSCs occurred after cryopreservation. Tested for their ability to improve pregnancy outcomes in mouse models of premature ovarian failure, placental MSCs were shown to restore fertility via the paracrine mechanism, with better therapeutic effects achieved using native rather than cryo cell populations. Overall, the study demonstrated that cryopreservation could be a valid option for long-term storage of placental tissue, maintaining the possibility of isolating placental MSCs with therapeutic potential. Using a rat model of full-thickness skin wounding, Zhang et al. assessed the comparative therapeutic potential of placental MSCs and their secreted exosomes in wound healing. In particular, both cells and derived exosomes stimulated the regeneration of cutaneous appendages (hair follicles and sebaceous glands), decreasing collagen I and increasing collagen III expression, as well as sustaining angiogenesis in the healed skin. Moreover, the effects of placental MSCs and their secreted exosomes were demonstrated to be mechanistically related to the downregulation of the Yes-associated protein (YAP) signaling pathway and the resulting inhibition of the activation of Engrailed-1 (EN1), which mediates the scarring process.

Interestingly, Tan et al. provided preclinical evidence of the immunomodulatory activity of bone-marrow-derived MSCs in a mouse model of Influenza A virus (IAV)-induced acute lung injury (ALI). Following the intravenous administration of MSCs to affected animals, granulocytic myeloid-derived cells, B cells (ASB), and T cells (CD8^+^) were recruited from circulation to the site of infection, with a significant reduction of immune cell infiltration into bronchoalveolar space. Additionally, a significantly higher proportion of the monocyte population with the M2 phenotype (CD206) was detected into the blood of MSC-treated animals. However, the immunomodulatory activity exerted by MSCs did not lead to any reduction in viral titer or animal mortality, probably because of MSC susceptibility to IAV infection, which would limit their therapeutic effects.

Exploring novel aspects of the osteogenic potential of bone-marrow-derived MSCs, Haddouti et al. designed a specialized setup to expose cells to shock waves as a non-invasive and effective therapy for treatment of musculoskeletal disorders. MSC responses to shock waves were analyzed, considering their viability, proliferation behavior, cytokine secretion, and osteogenic differentiation potential *in vitro*. Osteogenic differentiation was not significantly affected by a single shock wave application (SWA), but was increased four-fold after repeated sessions of SWA over three consecutive days. Moreover, repeated SWA triggered significant downregulation of type I collagen (COL1A1), upregulation of runt-related transcription factor 2 (RUNX2), and an increase in osteocalcin (OCN) in MSCs. Overall, the study was able to determine the SWA conditions that better enhanced MSC differentiation toward the osteogenic lineage *in vitro*, paving the way for the improvement of extracorporeal shock wave treatment in the clinical setting.

Finally, two review articles considered the clinical application of MSCs to treat neurological disorders and intervertebral disc degeneration. Specifically, Isakovic et al. examined *in vitro* and *in vivo* research clarifying the mechanisms of action of MSCs, as well as reviewing the findings of past and ongoing clinical trials involving MSC treatment for Parkinson’s and Alzheimer’s disease, ischemic stroke, glioblastoma multiforme, and multiple sclerosis. The study highlighted the fact that most of the research in the field supports the safety and efficacy of MSC-based therapy, with some adverse effects being recognized, especially in relation to possible differentiation into undesirable cell types, tumor-promoting ability, and initiation of an uncontrolled immune response. To overcome these limitations, the secretory capabilities of MSCs are now being investigated further and further, focusing on the therapeutic potential of MSC-derived exosomes and extracellular vesicles (EVs). When tested in clinical trials, MSC-based therapy has shown variable outcomes: functional recovery has been reported for neural pathologies such as multiple sclerosis, Alzheimer’s disease, and ischemic stroke, while no clear beneficial effect has yet been demonstrated for glioblastoma or Parkinson’s disease. Conversely, positive clinical outcomes were reported by Zhang et al. in their systematic review and meta-analysis of trials evaluating the efficacy of MSCs against intervertebral disc (IVD) degeneration in patients with lumbar discogenic pain. In particular, administration of MSCs in these patients provided pain relief and significantly improved scores on the Oswestry Disability Index, lowering the risk of adverse events and reoperation rates.

In conclusion, both experimental studies using animal models and clinical trials generally support the safety and efficacy of MSC-based therapies for tissue regeneration. However, many aspects still remain unexplored, requiring additional research to assess the risks associated with cell-based *versus* cell-free treatments, and to reduce outcome variability related to MSC heterogeneity.

## References

[B1] Al-Ghadban S. Artiles M. Bunnell B. A. (2022). Adipose stem cells in regenerative medicine: looking forward. Front. Bioeng. Biotechnol. 9, 837464. 10.3389/fbioe.2021.837464 35096804 PMC8792599

[B2] Antoniadou E. David A. L. (2016). Placental stem cells. Best. Pract. Res. Clin. Obstet. Gynaecol. 31, 13–29. 10.1016/j.bpobgyn.2015.08.014 26547389

[B3] Banerjee A. Rowlo P. Jothimani G. Duttaroy A. K. Pathak S. (2022). Wnt signalling inhibitors potently drive trans-differentiation potential of mesenchymal stem cells towards neuronal lineage. J. Med. Biol. Eng. 42, 630–646. 10.1007/s40846-022-00730-7

[B4] Barbon S. Rajendran S. Bertalot T. Piccione M. Gasparella M. Parnigotto P. P. (2021). Growth and differentiation of circulating stem cells after extensive *ex vivo* expansion. Tissue Eng. Regen. Med. 18, 411–427. 10.1007/s13770-021-00330-7 33625723 PMC8169750

[B5] Barbon S. Stocco E. Grandi F. Rajendran S. Borean A. Pirola I. (2018). Biofabrication of a novel leukocyte-fibrin-platelet membrane as a cells and growth factors delivery platform for tissue engineering applications. J. Tissue Eng. Regen. Med. 12, 1891–1906. 10.1002/term.2713 29956492

[B6] Barbon S. Stocco E. Rajendran S. Zardo L. Macchi V. Grandi C. (2022). *In vitro* conditioning of adipose-derived mesenchymal stem cells by the endothelial microenvironment: modeling cell responsiveness towards non-genetic correction of haemophilia A. Int. J. Mol. Sci. 23, 7282. 10.3390/ijms23137282 35806285 PMC9266329

[B7] Di Liddo R. Aguiari P. Barbon S. Bertalot T. Mandoli A. Tasso A. (2016). Nanopatterned acellular valve conduits drive the commitment of blood-derived multipotent cells. Int. J. Nanomedicine 11, 5041–5055. 10.2147/IJN.S115999 27789941 PMC5068475

[B8] Di Liddo R. Paganin P. Lora S. Dalzoppo D. Giraudo C. Miotto D. (2014). Poly-ε-caprolactone composite scaffolds for bone repair. Int. J. Mol. Med. 34, 1537–1546. 10.3892/ijmm.2014.1954 25319350

[B9] Dzobo K. (2021). Recent trends in multipotent human mesenchymal stem/stromal cells: learning from history and advancing clinical applications. OMICS 25, 342–357. 10.1089/omi.2021.0049 34115524

[B10] Han Y. Li X. Zhang Y. Han Y. Chang F. Ding J. (2019). Mesenchymal stem cells for regenerative medicine. Cells 8, 886. 10.3390/cells8080886 31412678 PMC6721852

[B11] Jothimani G. Di Liddo R. Pathak S. Piccione M. Sriramulu S. Banerjee A. (2020). Wnt signaling regulates the proliferation potential and lineage commitment of human umbilical cord derived mesenchymal stem cells. Mol. Biol. Rep. 47 (2), 1293–1308. 10.1007/s11033-019-05232-5 31853765

[B12] Kuhbier J. W. Weyand B. Radtke C. Vogt P. M. Kasper C. Reimers K. (2010). Isolation, characterization, differentiation, and application of adipose-derived stem cells. Adv. Biochem. Eng. Biotechnol. 123, 55–105. 10.1007/10_2009_24 20091288

[B13] Kulus M. Sibiak R. Stefańska K. Zdun M. Wieczorkiewicz M. Piotrowska-Kempisty H. (2021). Mesenchymal stem/stromal cells derived from human and animal perinatal tissues-origins, characteristics, signaling pathways, and clinical trials. Cells 10, 3278. 10.3390/cells10123278 34943786 PMC8699543

[B14] Li N. Gao J. Mi L. Zhang G. Zhang L. Zhang N. (2020). Synovial membrane mesenchymal stem cells: past life, current situation, and application in bone and joint diseases. Stem Cell Res. Ther. 11, 381. 10.1186/s13287-020-01885-3 32894205 PMC7487958

